# Supplementary material for: Identification of the Abscisic Acid-, Stress-, and Ripening-Induced (ASR) Family Involved in the Adaptation of Tetragonia tetragonoides (Pall.) Kuntze to Saline–Alkaline and Drought Habitats
Source: Int J Mol Sci. 2023 Oct 31;24(21):15815. doi: 10.3390/ijms242115815 (PMC10650104; doi:10.3390/ijms242115815)
Supplement: Supplementary file 1 [file ijms-24-15815-s001.zip › ijms-2686278-supplementary.pdf]

Supplementary Material Information

Identification of the *Abscisic Acid-, Stress-, and Ripening-Induced* (*ASR*) Family Involved in the Adaptation of *Tetragonia tetragonoides* (Pall.) Kuntze to Saline–Alkaline and Drought Habitats

Hao Liu [1,2,3], Qianqian Ding [1,2], Lisha Cao [1,2,3], Zengwang Huang [1,2], Zhengfeng Wang [1,3,4], Mei Zhang [1,5,*] and Shuguang Jian [3,6,*]

1 Guangdong Provincial Key Laboratory of Applied Botany, South China Botanical Garden, Chinese Academy of Sciences, Guangzhou 510650, China
2 University of Chinese Academy of Sciences, Beijing 100039, China
3 Key Laboratory of Vegetation Restoration and Management of Degraded Ecosystems, Center for Plant Ecology, Core Botanical Gardens, Chinese Academy of Sciences, Guangzhou 510650, China
4 Southern Marine Science and Engineering Guangdong Laboratory (Guangzhou), Guangzhou 511458, China
5 Center of Economic Botany, Core Botanical Gardens, Chinese Academy of Sciences, Guangzhou 510650, China
6 CAS Engineering Laboratory for Vegetation Ecosystem Restoration on Islands and Coastal Zones, South China Botanical Garden, Chinese Academy of Sciences, Guangzhou 510650, China
* Correspondence: zhangmei@scbg.ac.cn (M.Z.); jiansg@scbg.ac.cn (S.J.)

Table S1. The obtained *TtASR*s' nucleotide and protein sequences information in this study.

Table S2. Primers information used in this study.

Table S3. Summary of the *cis*-regulatory elements identified in the promoter regions of *TtASR* genes.
